# Supplementary material for: Simultaneous Irradiation with UV-A, -B, and -C Lights Promotes Effective Decontamination of Planktonic and Sessile Bacteria: A Pilot Study
Source: Int J Mol Sci. 2023 Aug 18;24(16):12951. doi: 10.3390/ijms241612951 (PMC10454392; doi:10.3390/ijms241612951)
Supplement: Supplementary file 1 [file ijms-24-12951-s001.zip › ijms-2541118-supplementary.pdf]

Simultaneous Irradiation with UV-A, -B, and -C Lights Promotes Effective Decontamination of Planktonic and Sessile Bacteria: A Pilot Study

Andrea Bosso [1,*], Francesca Tortora [1], Rosanna Culurciello [1], Ilaria Di Nardo [1], Valeria Pistorio [2], Federica Carraturo [1,3], Andrea Colecchia [4], Rocco Di Girolamo [5], Valeria Cafaro [1], Eugenio Notomista [1], Raffaele Ingenito [6] and Elio Pizzo [1,7,*]

[1] Department of Biology, University of Naples Federico II, 80126 Naples, Italy
[2] Centre de Recherche Saint-Antoine (CRSA), Sorbonne University, Inserm, 75012 Paris, France
[3] Hygiene Laboratory, Centro Servizi Metrologici e Tecnologici Avanzati (CeSMA), University of Naples Federico II, Corso Nicolangelo Protopisani, 80146 Naples, Italy
[4] Physics Department "Ettore Pancini", University of Naples Federico II, 80126 Naples, Italy
[5] Department of Chemical Sciences, University of Naples Federico II, 80126 Naples, Italy
[6] Naturamla S.R.L., 00195 Rome, Italy
[7] Centro Servizi Metrologici e Tecnologici Avanzati (CeSMA), University of Naples Federico II, 80126 Naples, Italy
* Correspondence: **Corresponding Authors**: andrea.bosso@unina.it, +39 081679129, elipizzo@unina.it +39 081679151

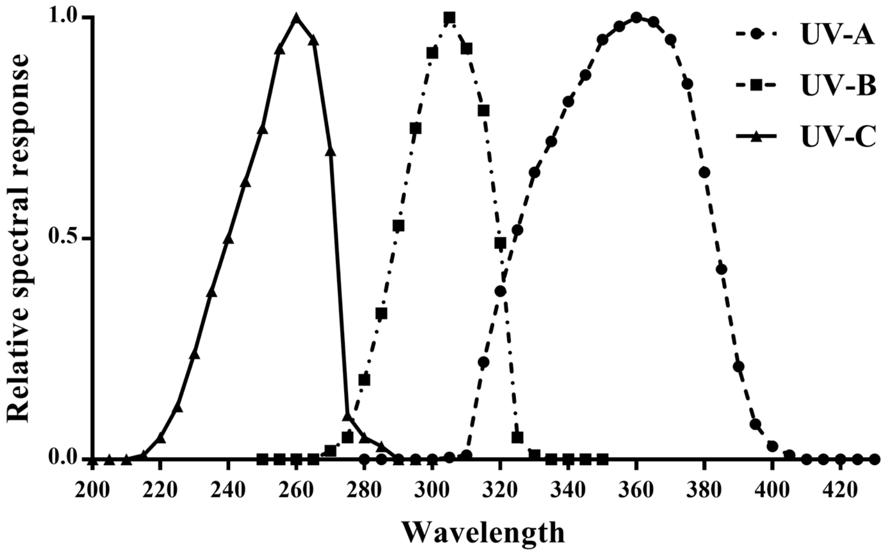

Figure S1. Relative spectral response of the device equipped with three UV channels.

Table S1. Technical data of UV-ABC lamp.

**TECHNICAL DATA**	**UVC + UVB + UVA**
Power **UVC**	2x 120 W
Power **UVB**	60 W
Power **UVA**	60 W
Power **LED**	45 W
Color Temperature	4000 K
Lumen	6720 (140 Im/W)
Dimensions	1200 x 170 x 55 mm
Weigth	4,0 Kg

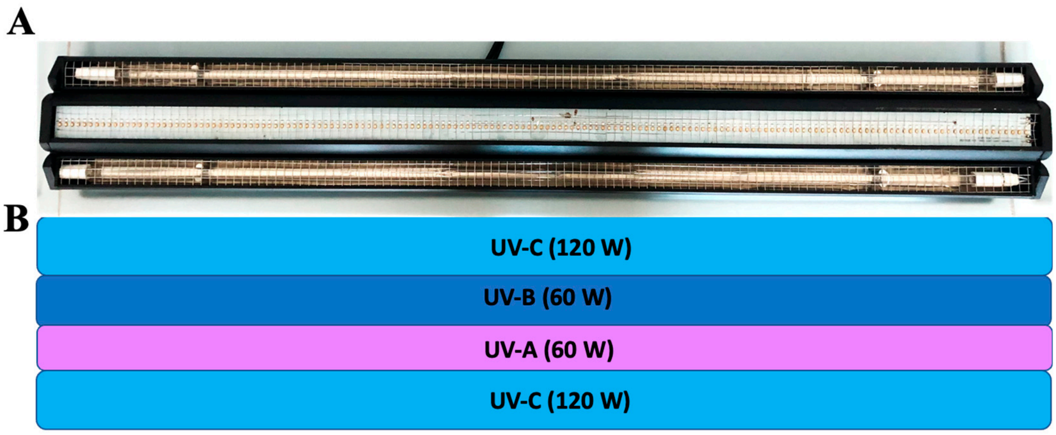

Figure S2. (A) Picture of the UV-lamp described in the text, (B) its scheme of light sources and power.

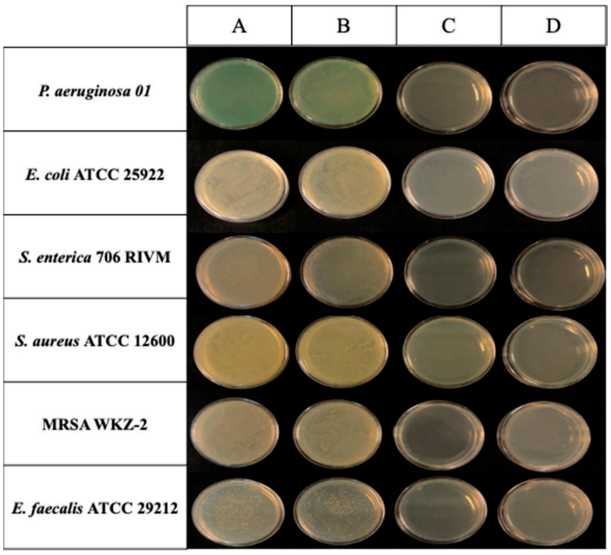

Figure S3. Analysis of UV treatment on solid medium before and after seeding of bacteria. (A) Control: bacteria were seeded on solid media and grown at 37°C for 16 hours. (B) the solid medium was pretreated with UV light for 5 minutes and then the bacteria were plated and let grow. (C) The solid medium was seeded with bacteria and then treated with a UV lamp for 5 minutes. (D) The solid medium was pretreated with UV for 5 minutes in the absence of bacteria, then the bacteria were seeded, and the plate was again treated with UV for 5 minutes.

Table S2. Irradiance and Energy dose of UV-C lamp measured from a distance of 60 cm.

	UV-C (240 W)	
**Time (minutes)**	**Irradiance (mW/cm^2)**	**Energy Dose (mJ/cm^2)**
**0**	**0,2**	**12**
**5**	**0,371**	**111,3**
**30**	**0,189**	**340,2**
**60**	**0,18**	**648**
**180**	**0,16**	**1728**

Table S3. Technical data of UV-C lamp.

**TECHNICAL DATA**	**HF -UVC**
Power **UVC**	240 W
Power **LED**	45 W
Color Temperature	4000 K
Lumen	6720 (140 Im/W)
Dimensions	1200 x 170 x 55 mm
Weigth	4,0 Kg

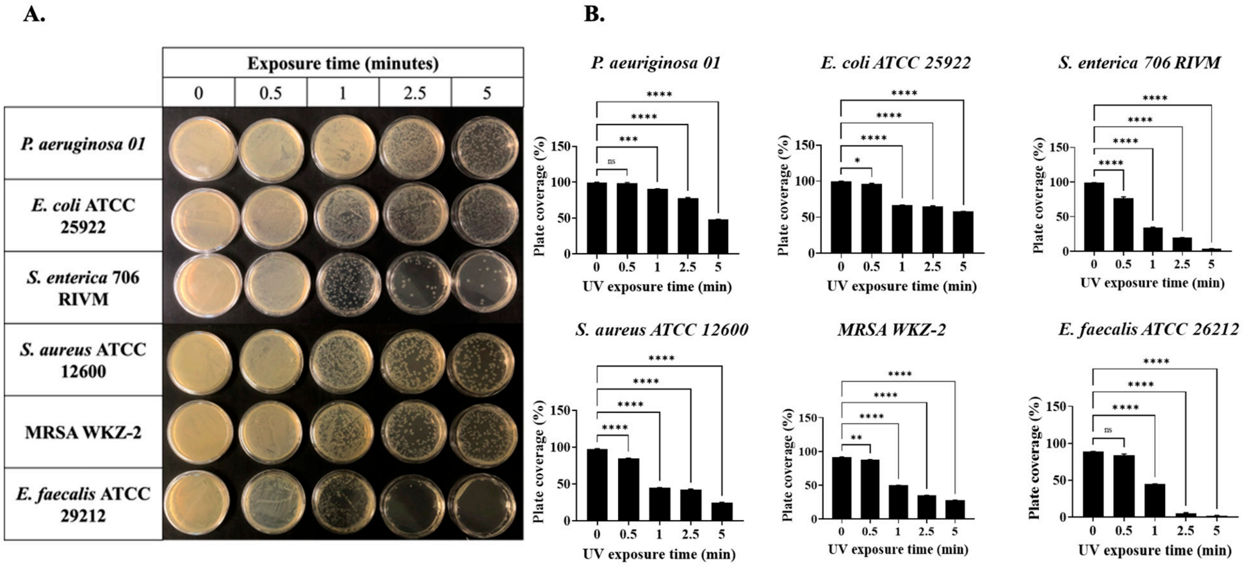

Figure S4. Effects of UV-C radiations on six different bacterial strains plated on Petri dishes. **(A)** Plates seeded with Gram-negative and Gram-positive strains were exposed to UV radiations for 0.5, 1, 2.5 and 5 minutes; **(B)**. Analysis of percentage of plate coverage after UV exposure for 0.5, 1, 2.5 and 5 minutes.

Table S4. Results of the on-field tests for the evaluation of the microbicidal effect of 5, 60 and 180 minutes of UV exposures performed on a PVC surface after natural contamination.

UV lamp tests performed on naturally contaminated PVC surface				
**Analyzed parameter** (Results expressed in CFU/100 cm^2)	**Before the treatment**	**5 min treatment**	**60 min treatment**	**180 min treatment**
**Total Bacterial Count at 22°C**	132	20	8	4
**Total Bacterial Count at 37°C**	32	4	0	0
**Molds and yeasts**	108	16	12	8

Microbial die-off rates following the treatment with UV lamp on naturally contaminated PVC surface			
**Analyzed parameter** (Results expressed in CFU/100 cm^2)	**Die-off rate**		
	**5 minutes**	**60 minutes**	**180 minutes**
**Total Bacterial Count at 22°C**	84.85	93.94	96.97
**Total Bacterial Count at 37°C**	87.50	100.00	100.00
**Molds and yeasts**	85.19	88.89	92,59

Table S5. Results of the on-field tests for the evaluation of the microbicidal effect of 5, 60 and 180 minutes of UV exposures performed on rough ceramic tile after natural contamination.

UV lamp tests performed on naturally contaminated rough ceramic tile				
**Analyzed parameter** (Results expressed in UFC/100 cm^2)	**Before the treatment**	**5 min treatment**	**60 min treatment**	**180 min treatment**
**Total Bacterial Count at 22°C**	192	28	8	4
**Total Bacterial Count at 37°C**	128	8	4	2
**Molds and yeasts**	48	8	0	0

Microbial die-off rates following the treatment with UV lamp on naturally contaminated rough ceramic tile			
**Analyzed parameter** (Results expressed in UFC/100 cm^2)	**Die-off rate**		
	**5 minutes**	**60 minutes**	**180 minutes**
**Total Bacterial Count at 22°C**	85.42	95.83	97.92
**Total Bacterial Count at 37°C**	93.75	96.88	98.44
**Molds and yeasts**	83.33	100.00	100.00
